# Effect of 9 weeks continuous vs. interval aerobic training on plasma BDNF levels, aerobic fitness, cognitive capacity and quality of life among seniors with mild to moderate Alzheimer’s disease: a randomized controlled trial

**DOI:** 10.1186/s11556-019-0234-1

**Published:** 2020-01-06

**Authors:** Lievyn Enette, Thomas Vogel, Sylvie Merle, Anna-Gaelle Valard-Guiguet, Nathalie Ozier-Lafontaine, Remi Neviere, Claudia Leuly-Joncart, Jean Luc Fanon, Pierre Olivier Lang

**Affiliations:** 1 Research Laboratory Mitochondria, Oxidative stress and muscle resistance (MSP, EA-3072), Department of Physiology, Faculty of Medicine, Strasbourg University, Résidence La Yole, bat. B L’Etang Z’abricot, 97200 Strasbourg, France; 2 Department of geriatric, University Hospital, Strasbourg, France; 3 Methodology and biostatistics Unit (DRCI), University Hospital Centre of Martinique, Fort de France, France; 4 The Caribbean reference center for rare neuromuscular and neurologic diseases (CeRCa), University Hospital Centre of Martinique, Fort de France, France; 5 Department of Functional Exploration and Non-Invasive Cardiology, University Hospital Centre of Martinique, Fort de France, France; 6 Department of cardiology, University Hospital Centre of Martinique, Fort de France, France; 7 Department of Geriatric and Gerontology, University Hospital Centre of Martinique, Fort de France, France; 8 Clinic of Montchoisi, Lausanne, Switzerland

**Keywords:** Aerobic exercise, aerobic fitness, BDNF, Alzheimer’s disease, cognitive performance, older adults

## Abstract

**Background:**

Evidence suggests that aerobic-type training confers physical benefits and appears to contribute positively to brain health. This study aims to compare the effect of 9-weeks continuous (CAT) to interval aerobic training (IAT) on brain derived neurotrophic factor (BDNF) plasma level, aerobic fitness, cognitive performance, and quality of life among senior with Alzheimer’s disease (AD).

**Methods:**

52 participants were randomly allocated into three groups (CAT *n* = 14; IAT *n* = 17; and Controls *n* = 21). CAT and IAT consisted of 18 sessions of 30-min cycling, twice a week, over 9 weeks. During the same period, controls were engaged in interactive information sessions. Plasma BDNF level; aerobic fitness parameters (Metabolic equivalent task - METs; Maximal Tolerated Power – MTP); functional capacities (6-Minute Walk Test - 6MWT); cognitive performance (Mini Mental State Examination; Rey auditory verbal learning test; and digit span test) and quality of life (Quality Of Life of Alzheimer’s Disease scale - QoL-AD) were measured in all participants at baseline and 9 weeks later. A third plasma BDNF level was quantified following a 4 weeks detraining.

**Results:**

No significant change was measured in terms of plasma BDNF level and cognitive performance after interventions, in all groups compared to baseline. After 9 weeks, CAT and IAT significantly improved aerobic fitness parameters compared to controls (METs: + 0.6 and + 1.0 vs. + 0.4; MTP: + 16 watts and + 20 watts vs. + 10 watts; and functional capacities (6MWT: + 22 m and + 31 m vs. -40 m). Compared to controls, QoL-AD after CAT was improved (+ 2 points; *p = 0.02*).

**Conclusions:**

Neither aerobic exercise modalities significantly modified plasma BDNF levels and cognitive performances. CAT and IAT enhanced aerobic fitness and functional capacities in AD patients and CAT their QoL.

**Trial registration:**

ClinicalTrials.gov website (NCT02968875); registration date: 7 September 2016. “Retrospectively registered”.

## Introduction

Alzheimer’s disease (AD) is the most prevalent cause of cognitive impairment among the aging and aged population [1]. The hallmarks of this neurodegenerative disease are extracellular deposition of amyloid-βeta (Aβ) plaques, intracellular neurofibrillary tangles (i.e., aggregates of hyper phosphorylated tau protein), and the progressive decline of the cholinergic activity in basal forebrain neurons [2]. By primarily affecting the hippocampus, the resulting cell death and brain atrophy progressively impact long-term memory and learning capacities, and at later stages, the patients’ functional abilities to perform the simplest tasks of the daily life [3]. To date, only symptomatic pharmacological therapies modulating the acetylcholine or glutamate activity are available and none of them significantly slows down the course of the disease. Hence, developing strategies that could potentially maintain the highest possible cognitive performance, functional abilities, and quality of live (QoL) is important for altering AD progression [4]. Exercise including aerobic-type training such as continuous aerobic training (CAT) or interval aerobic training (IAT), is one valuable intervention to improve the brain health (i.e., structure and functions) in humans [5–7]. CAT is defined as continuous training performed at moderate intensity while IAT consists of periods high-intensity exercise alternated by period of relative recovery. Thus, as a complementary mean to the control of all common cardiovascular risk factors which are also implicated in AD physiopathology, improvement of cardiorespiratory fitness parameters is also associated with higher hippocampal volume, enhancement in functional capacities together with personal development [8, 9]. In AD, studies suggest that aerobic-type training might attenuate brain atrophy and symptoms by modulating the gene expression of nerve growth factor and neurotrophic factor. Those factors are important for neurogenesis, synaptogenesis, and neurotransmission [10–12]. By this ways, aerobic-type training might slow down the production of Aβ plaques and oxidative stress, which have deleterious effects on neurons activity in AD [13–15]. Of all neurotrophic factors, brain derived neurotrophic factor (BDNF) seems to be one of the most susceptible to be modulated by aerobic-type exercise [16, 17]. Member of the neurotrophin family, it is a growing and survival factor regulating the development and the maintenance of functional phenotype of neuronal cells, and the controls of the synaptic function and neuronal plasticity [18]. Acting via the tropomyosin-related kinase B (TrkB), BDNF has thus emerged as an important mediator of the memory process, the adaptability of the synaptic transmission, and more globally of the brain plasticity [18]. Although still unclear, several potential explanations have been suggested to explain the biological mechanism between aerobic-type exercise and BDNF pathways [19]. Warnn et al. reported that FNDC5 protein is released during exercise from skeletal muscle, inducing BDNF from hippocampus [20]. Other studies support that the vascular endothelium could be a other candidate source of syntheses of peripheral BDNF during exercise [21, 22]. However, the exact impact of the intensity level as the underlying regulator explaining some of the benefits of functional and mental health outcomes is still controversial [23, 24].

Although evidence of the benefits of aerobic-type training on cardiorespiratory, cognitive performance, and QoL are consistent among healthy individuals (including seniors) [25], the respective impact of intensity (moderate vs. vigorous) and type (CAT vs. IAT) in mild to moderate AD patients remain unclear [26, 27]. Furthermore, the effect intensity and type of aerobic-type training modulates the BDNF level in this population has not yet been answered [28, 29]. Hereinafter are presented the results of the MARAE study (“*Maladie d’Alzheimer et Réentrainement À l’Éffort*” in French language) which analyzes the effect of CAT and IAT respectively on the plasma BDNF level, aerobic fitness parameters, walking capacity, cognitive performance, and QoL in seniors with mild to moderate AD. The feasibility of such training programs in this population was also an outcome.

## Materials and methods

### Study design

**Fig. 1:**
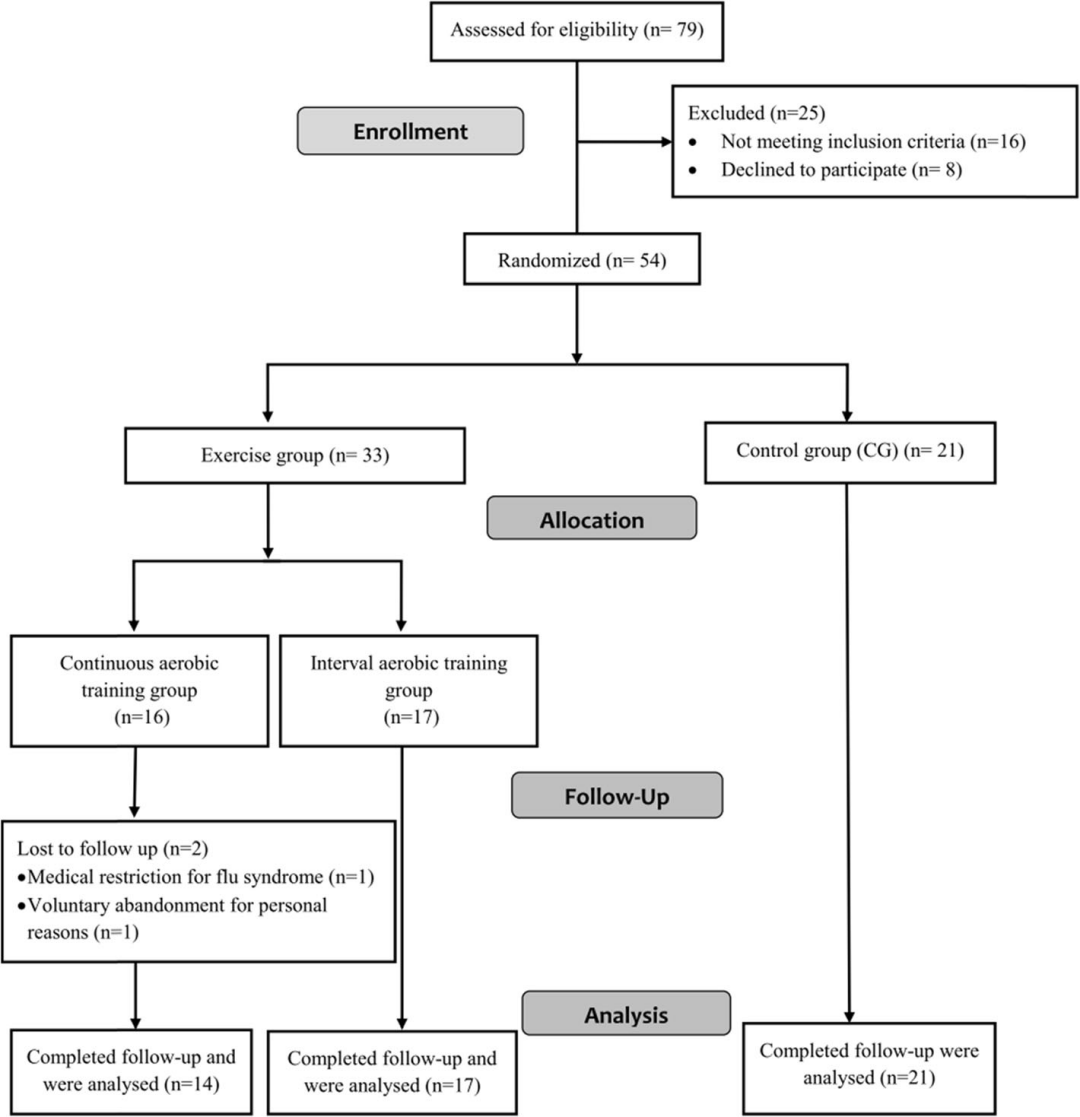
Consort flow diagram of inclusion, randomization and follow-up including reasons for drop-out

**Fig. 2:**
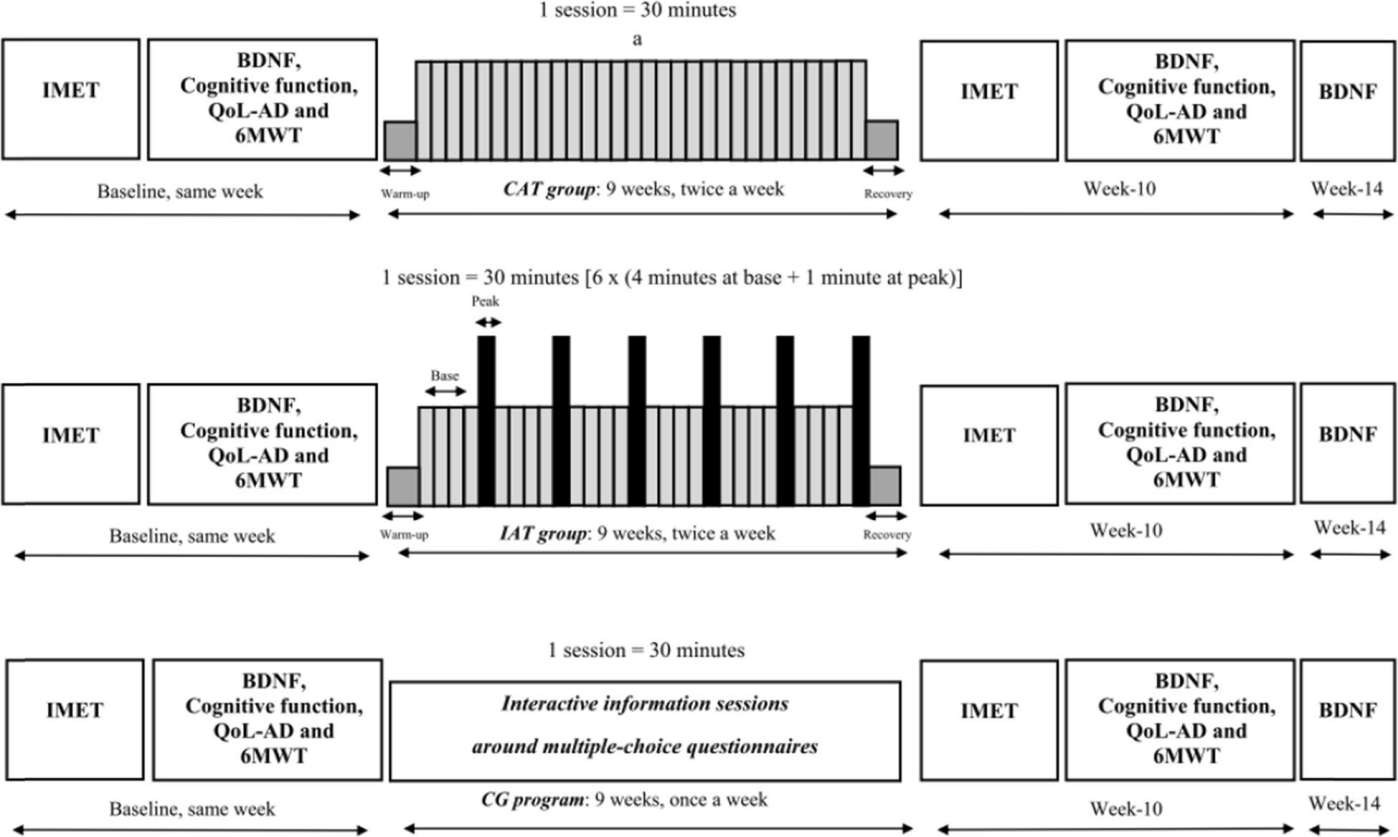
Study design and visual presentation of CAT, IAT and CG program. a = watts equivalent to 70% of HR_max_ or 50% of MTP. Base = watts equivalent to 60% of HR_max_. Peak = watts equivalent to 80% of HR_max_ or MTP – 10 watts. Abbreviations: IMET, Incremental Maximal Exercise Test; BDNF, Brain Derived Neurotrophic Factor; QoL-AD, Quality of life of Alzheimer disease; 6MWT, 6 Minutes walking – test

The MARAE study was a randomized controlled trial conducted at the memory clinic of the University Hospital Centre of Martinique (Fort de France, France). All participants were recruited from April 19, 2016 to July 31, 2017 following an invitation to take part in the research on the benefits of exercise on general and mental health. Once included, participants were allocated to a training or control group (CG) with 2:1 ratio. Participants allocated to the training were secondarily randomized with a 1:1 ratio to either CAT or IAT program (Fig. 1). The allocation between the three groups was balanced as much as possible by considering blocks randomization of size 12. During the course of the study, members of the MARAE team supervised participants during training and CG period, and experienced specialists collected the primary (i.e., plasma BDNF concentration expressed in pg/ml) and secondary outcomes (i.e., aerobic fitness parameters, functional capacities, cognitive functions. and the participants’ QoL). All study outcomes were measured and collected blind to the allocation group at baseline, after 9 weeks intervention period (referred as week-10) and only for plasma BDNF concentration, a third blood sample was collected 4 weeks after a detraining period, (referred as week-14 – Fig. 2). The experimental protocol was approved by the local ethic committee (2015A00567–42 – www.cpp-soom3.u-bordeaux2.fr) and was registered on the ClinicalTrials.gov website (NCT02968875).

### Participants

In order to detect a significant 30% difference between post- and pre-training period in the plasma BDNF level (primary outcome) between training and control groups with a statistic power of 90%, the enrolment goal of 20 participants by study group was calculated, taking into account the probability of 10% of dropout [30].

Volunteers among patients followed-up at the memory clinic were invited to participate without financial incentives. To be eligible, they had to be aged 60 years or over, functionally independent, able to exercise at moderate to high intensity level on cycle ergometers, and diagnosed with a mild to moderate AD according to the criteria of the revised version of the DSM (Diagnostic and Statistical Manual) fourth edition. At the time of inclusion, the Mini Mental State Examination (MMSE) score had to be 16/30 or over [31, 32]. All volunteers, thus identified, completed a medical visit including a physical examination with the measurement of anthropometric parameters, resting heart rate (HR), systolic blood pressure (SBP) and diastolic blood pressure (DBP). A 12-lead electrocardiogram was also recorded. Body height was determined with a measuring rod, and total body weight and percentage of body fat were measured with Tanita weight and by bio-impedance respectively (Tanita®, SC-240MA). Body mass index (BMI) was calculated as weight divided by height squared (kg/m^2^) and participants classified according to the World Health Organization classification as underweight (< 18.5 kg/m^2^), normal weight (18.5 to 24.9 kg/m^2^), overweight (25 to 29.9 kg/m^2^), and obese class I (30 to 34.9 kg/m^2^) and II or over (≥35 kg/m^2^). With the complete review of the current and past medical history, all conditions that contra-indicated aerobic training were identified (e.g., uncontrolled hypertension, severs musculoskeletal/musculotendinous disorders or fibromyalgia). In addition, individuals with sever cognitive impairment, undergoing chemotherapy for cancer, or suffering from any acute infection were not enrolled as well as people already engaged in any aerobic training program. The medication regimen was also recorded but not any specific treatment was considered as an inclusion or non-inclusion criteria.

During the medical interview, volunteers received information and instructions about the MARAE study, and the volunteer and his/her caregiver signed an informed consent before final inclusion. At the time of inclusion, volunteers were also requested to maintain their usual diet habits all along the duration of the study period. Participants could be excluded from the study protocol over time in case of the occurrence of any chest pain, hypertensive response to exercise (i.e.*,* a difference between peak and baseline SBP of at least 60 mmHg in men and 50 mmHg in women during exercise testing, or SBP exceeding 210 mmHg in men and 190 mmHg in women [33]*,* any rhythm disorders, ST segment deviation, and/or respiratory problems during the baseline IMET.

### Primary outcome: circulating plasma BDNF level

Blood samples were collected by venipuncture into EDTA tubes, at rest for at least 5 min and between 08.00 and 09.00 am after overnight fasting at baseline, at week-10 (i.e.*,* after, CAT, IAT, or information sessions for CG) and at week-14, following a 4 weeks detraining. Immediately, tubes were placed on ice, and centrifuged for 20 min at 3000 rpm at 8 °C. The plasma was then collected into sterile 500 μL microtubes and frozen at − 80 °C until analyses (from 1 to 18 month according the participant). Plasma BDNF levels were measured by Enzyme-Linked Immunosorbent Assay (ELISA – BDNF Emax® Immunoassay System, Promega, Madison, WI, USA) with appropriate dilution. All samples and standards were run in duplicate. When the BDNF value was out of the standard curve, a new measurement with a ¼ dilution was then run in duplicate. The means of the duplicates were used for subsequent statistical analyses. Each standard curve had a coefficient of determination about r^2^ = 0.999 [see Additional file 1 for further details].

### Secondary outcomes

#### Aerobic fitness parameters

In the three study groups, an incremental maximal exercise tests (IMET) supervised by an experienced cardiologist was carried out at baseline and at week-10 (Fig. 2). Before the IMET, HR, brachial SBP and DBP were measured seating after a rest of at least 5 min. Participants were then installed on an upright electronically braked cycle ergometer (Ergo-Line GmbH&Co KG 800 s, Bitz, Germany). The IMET was carried out in an air-conditioned room (22.0 ± 0.5 °C), two hours after a light lunch. After a 2-min warm-up period at 10–25 watts, charge increments of 10 watts min^− 1^ were used with at a pedaling frequency of > 60 round per minute until to exhaustion, completed in 6–12 min [34]. IMET was terminated either because of cardiovascular exhaustion (HR > 85% of maximal HR theoretical (HR_max_ = 220-minus age)) and/or leg fatigue associated with a perceived exhaustion > 17 on Borg scale 6–20, requiring the end of the test [35]. Aerobic fitness capacity was expressed in maximal Metabolic Equivalent of Task (METs) and estimated based on the Maximal Tolerated Power (MTP) measured in watt using the following formula from the American College of Sports Medicine (METs = (12,3 x Watts) + (3,5 x weigh) / (weigh × 3,5)) [36]. The MTP, METs and maximal HR (defined as heart rate peak – HR_peak_) were collected at the end of the test. During the IMET, HR was continuously monitored (Cardiovit CS-200, Schiller AG, Baar, Switzerland). For participants enrolled in the CAT and IAT groups, workloads determined from a percentage of HR_max_ measured during the IMET, were considered to set the initial intensity target values for the first training sessions.

#### Functional capacities

A physiotherapist conducted a 6-min walking test (6MWT) at baseline and at week-10, in the CAT, IAT and CG (Fig. 2). The distance walked in meters was considered to evaluate the effect of the study intervention on functional capacities. The 6MWT was performed indoors along a flat firm surface (i.e.*,* a 40 m hallway) according to standard recommendations [37].

#### Cognitive performance tests

In the three groups, an experienced neuropsychologist assessed the cognitive performance at baseline and at week-10 (Fig. 2). Validated tests were then considered to assess global cognition functioning (MMSE) [38, 39], episodic memory (Rey Auditory Verbal Learning Test - RAVLT) [40] and working memory (Digit span test) [41] according to standard instructions. To measure the impact of the study interventions, the scores of the different sub-sets of the MMSE (i.e., orientation, registration and free recall, attention and calculation, language, and visual construction) were also analyzed individually.

#### Quality of life assessment

Finally, the QoL-AD scale [42] was used to assess the health-related QoL of all participants at baseline and at week-10. QoL-AD scale is a brief, 13-item measure, designed to provide both individual and proxy report which were calculated separately. This tool explores thirteen domains of the health-related QoL (i.e., physical health, energy, mood, living situation, memory, family, marriage, friends, self as a whole, ability to do chores, ability to do things for fun, money, and life as a whole). Each item was answered according to a Likert scale (from 1 – worst possible QoL to 4 – best possible QoL), for a total score between 13 (min) and 52 (max) [43]. According the standard instructions, to provide an assessment that may be more balanced than just either score alone, a single composite score combining the patient and caregiver ratings was calculated by multiplying the patient score by 2, adding the caregiver score and dividing the sum by 3 [42, 43].

### Assessment of the feasibility of the training programs

The feasibility of the training programs was evaluated according to (*i*) the adherence rate defined as the number of sessions undertaken compared to the number of session scheduled, (*ii*) the level of exercise intensity reached (i.e., watts and perceived exertion), and (*iii*) the progressive enhancement (i.e.*,* increase of workload over time). All adverse events such as muscles injuries, and muscle and joint pains occurring during the training program; and the number of participants dropped out and reasons why were also collected. Systematically, at the beginning of each session, all participants were questioned about the occurrence of any events since the last session and this until the end of the program.

### Additional data

Socio-demographic characteristics of each participant together with data concerning the health status, drugs, and the ability to perform Activity of Daily Living (ADL) and Instrumental Activity of Daily Life (IADL) were also collected at baseline. In addition, the presence/absence of a depressive disorder was systematically screened according to the geriatric depressive symptoms scale (GDS) [44]. The inflammatory profile of participants was also evaluated with serum C-reactive protein (CRP) as marker, at baseline. The serum CRP assay was carried out by a venous blood sample, into an EDTA tube, and analyzed with an automatic COBAS 6000 analyzer by a nephelometric method (Roche Diagnostics GmbH, Mannheim, Germany). The limit detection was 0.6 mg/L.

### The aerobic training groups (CAT and IAT)

As depicted by Fig. 2, CAT and IAT consisted of a 30-min cycling workout twice a week over 9 weeks (i.e. for 18 sessions in total) on an upright electronically braked cycle ergometer (Kettler GmbH & Co KG E5, Kleinblittersdorf, Germany). Each session was started with a 2-min warm-up and finalized with 1-min cool down. An experienced and trained physiotherapist supervised all the training sessions. HR was continuously monitored and automatically recorded (Polar H10, Kempele, Finland). SBP and DBP were also systematically measured (Omron M3 V4, Kyoto, Japan) before and after each session. In order to determine the appropriate intensity of CAT and IAT for each participant, we applied exercises protocols using either a fixed percentage of HR_max_ or a fixed percentage of workload of MTP for participants with low fitness levels. This approach recommended to improve and maintain health in older adults was based on the recommendations of the American College of Sports Medicine and American Heart Association but also on previous investigations that assessed the feasibility of aerobic-type training in AD [45–47]. More specifically for CAT, participants performed 30 min on ergocycles at a power level equivalent to 70% of their HR_max_ or 50% of MTP for participants who did not reach at least 85% of HR_max_, measured during the IMET. From the second session, whenever the physiotherapist observed a decrease of 5 to 10 beats in HR or perceived exertion from 1 point (Borg 6–20) for the same workload after each session, the intensity was reevaluated and increased by approximately 5 to 10 watts at the next session in order to maintain the prescribed intensity.

For IAT, participants exercised 6 × 1 minute at a workload equivalent to 80% of HR_max_ (for participants who did not reach at least 85% of HR_max_ during the IMET) or at a workload equal to MTP inferior to 10 watts for others (PEAK). This was combined with 4 min of active recovery at a workload equivalent to 60% HR_max_ between intervals (BASE). As in CAT, any decrease in HR from 5 to 10 beats or perceived exertion from 1 point (Borg 6–20) for the same workload after each session, from the second session, resulted in an up adjustment approximately of 5 to 10 watts of the intensity of the load at “BASE” and “PEAK” at the next session [48]. During the exercise, HR values measured at the 28th and 30th minutes were considered “target values” for the entire program.

### Interactive information sessions

In order to maintain their motivation [49], participants assigned to the CG were asked to attend interactive information sessions. They were also asked to not engage in any exercise training programs during all the study period (i.e. 14 weeks in total). Then, once a week, controls attended sessions detailing the health benefits of physical activity in seniors. These supervised sessions were structured with standardized multiple-choice questionnaires that participants had to discuss after together in an interactive way. In total, patients attended 9 sessions of 30-min each as shown in Fig. 2.

### Statistical analysis

Analyses were computed with STATA (version 15.0) and R (version 3.3.2) software with a significant threshold set at *p = 0.05*. Descriptive analyses were expressed for quantitative variables as median (Med), and interquartile range (IQR); for categorical variables, sample size was given. Specifically for BDNF values, the outlier-labeling rule was applied to define outliers in measures at baseline, at week-10 and at week-14. Observations that lie more than 2.2 times the interquartile range away from the nearest quartile were considered as outliers [50]. Two types of comparative analyses were computed. Thus, comparisons were computed between the three study groups (i.e.*,* CAT, IAT and CG) and within each study group for pre- and post-intervention outcome measures respectively. The primary outcome measure was BDNF plasma (pg/ml). Secondary outcomes were aerobic fitness parameters (METs, MTP); distance walked at the 6MWT; global MMSE scores and sub-tests, RAVLT, and Digit span scores; and total of composite QoL-AD scores. Participants’ characteristics at baseline were also compared according to the allocation group. Due to the small sample size and a majority of variables which did not meet the assumptions for normal distribution, we analyzed the data using non-parametric statistics. Thus, quantitative data were compared with Mann-Whitney or Kruskal-Wallis tests for inter-group comparisons and using the Wilcoxon test for paired samples for intra-group comparisons; for qualitative data the Fisher’s exact test was considered. Specifically, to analyze the impact of some potential confounders variables (i.e.*,* gender; age; MMSE score; aerobic fitness capacity in METs; intake of pro-cognitive treatment, storage duration of samples, intake of antidepressant drugs, chronic alcohol and/or tobacco consumption) on plasma BDNF values at baseline, a multivariable analysis of covariance (ANCOVA) was also considered. Chronic alcohol consumption was defined as consumption of more than two alcoholic beverages a day for men and one for women whereas smokers were defined as at least five cigarettes per day smoked since nine or more years [51, 52]. Additionally, a comparison of plasma BDNF level between AD at mild stage (MMSE ≥21) and moderate stage (MMSE < 21) was computed. A Pearson’s correlation coefficient was calculated to analyze the relationship between plasma BDNF level and cognitive performance according to MMSE, RAVLT, and digit span score.

## Results

Out of the 79 volunteers who were willing to participate in the study, 54 were finally enrolled whom 52 completed the study protocol and follow-up. Reasons for non-inclusion and withdrawal are presented in Fig. 1. Participants aged 77.9 ± 7.6 years (Min: 62 years; Max: 96 years) were for 31 assigned into one of the two training groups (CAT = 14; IAT = 17) and 21 were controls**.** Their characteristics at baseline are detailed in Table 1. No significant difference was measured between the 3 study groups except that individuals assigned into the two training groups were on average, lighter in body weight than controls due to a higher prevalence of underweight participants (*p = 0.05*). At baseline, individuals assigned into IAT group had a higher level of performance according to the METs value than controls (*p = 0.03*). Additionally, when participants (*n* = 52) were divided by disease stage (MMSE ≥21 = 18; MMSE < 21 = 34), plasma BDNF level was not significantly different, MMSE ≥21: 256.5 pg/ml, 106.9–449.7 pg/ml; MMSE < 21: 253.6 pg/ml, 121–509.7 pg/ml – *p = 0.71.*

**Table 1:** Baseline demographic and clinical characteristics of participants by group

Characteristic	CAT group, *n* = 14	IAT group, *n* = 17	Control group, *n* = 21
Age (years)	74 (68–83)	79 (75–82)	79 (75–84)
Female, *n*	11	11	11
Male, *n*	3	6	10
Height (cm)	167 (163–171)	163 (160–170)	170 (162–175)
Body weight(kg)	65 (60–71)	60 (52–67) ^a^	72 (60–77) ^a^
Fat mass (%)	30 (22–35)	23 (19–27)	26 (22–36)
BMI (kg/m^2^)	23 (21–26)	22 (20–24)	23 (21–26)
BMI category			
Underweight, *n*	1	2	0
Normal weight, *n*	9	13	13
Overweight, *n*	4	1	6
Obese, *n*	0	1	2
Nursing home residing, *n*	2	4	5
Home-living population, *n*	12	13	16
Education level (years)	10 (7–10)	7 (7–7)	7 (7–10)
Alzheimer duration (years)	2 (1–4)	2 (1–5)	6 (3–6)
Severity of dementia			
Mild; MMSE ≥21	5	2	11
Moderate; MMSE < 21	9	15	10
GDS, score	0.1 (0.2–0.5)	0.5 (0.6–1)	0.6 (0.3–0.9)
ADL, score	6 (5.5–6)	6 (5.9–6)	5.5 (4.6–6)
IADL, score	4 (3–6)	4 (3–6)	3.5 (2–5)
Medicine			
Antihypertensive treatment, *n*	8	12	13
Statins, *n*	2	4	2
Antidiabetic medication, *n*	2	4	4
Depression treatment, *n*	0	3	5
Anxiety treatment, *n*	1	2	2
Anti-psychotic, *n*	1	0	1
Anti – Alzheimer’s treatment			
Cholinesterase inhibitors, *n*	6	8	6
NMDA receptor antagonist, *n*	2	2	3
Comorbidities			
Charlson, score	1 (1–1)	2 (1–2)	1 (1–2)
Hypertension, *n*	8	12	13
Diabetes, *n*	2	4	4
Hypercholesterolemia, *n*	3	4	2
Inflammatory marker			
C-reactive protein _(mg/L)_	0.6 (0.6–1)	0.6 (0.6–1.3)	1 (0.6–2)
BDNFp _(pg/ml)_	194.9 (95.1–315)	353.8 (109–452.7)	254 (128.5–542.2)
METs	4.2 (3.2–4.9)	4.4 (3.9–4.9) ^a^	3.4 (2.9–4.2) ^a^
MTP	52 (42–60)	50 (50–66)	40 (35–70)
HR _peak_ (Beats per minute)	124 (116–141)	120 (113–126)	112 (106–120)
Measured HR _peak /_ HR _max_ (%)	87	85	81
RPE at HR_peak_	18 (17–18)	17 (17–18)	17 (17–18)
6MWT (meters)	470 (402–494)	430 (370–460)	420 (360–450)
MMSE score	18 (16–21)	18 (17–19)	21 (17–23)
RAVLT	22 (13–26)	21 (16–25)	19 (18–24)
Forward Digit Span	5 (3–6)	4 (4–5.3)	5 (4–7)
Backward Digit Span	2 (2–3)	3 (2–3)	3 (2–4)
QoL-AD composite	34 (32–36)	34 (32–35)	32 (29–34)

Median and range are provided unless otherwise indicated. ^a^: between group difference (*p < 0.05*)Abbreviations: *n* = Number; BMI = Body Mass Index; Underweight BMI < 18.5; Normal weight BMI = 18.5–24.9; Overweight BMI = 25–29; Obese BMI > 30; MMSE = Mini-Mental Status Exam (maximum score = 30); GDS = Geriatric disease scale: Score 0 to 4, score > 1 indicating high probability of depression; ADL = Activity of Daily Life, score 0 to 6 high score indicate more independence; IADL = Instrumental of Activity of Daily Life, score 0 to 8 high score indicate more independence; NMDA = N-methyl-D-aspartate; BDNFp: Plasma Brain derived neurotrophic factor; MET = Metabolic equivalent task; HR = Heart rate; RPE = Rating perceived exhaustion (Borg scale 6–20). 6MWT = 6 Minutes Walk Test; RAVLT: Rey Auditory Verbal learning Test, score 0 to 75 with higher score indicating better memory. QoL-AD: Quality of Life in Alzheimer’s Disease, total score 13 to 52 with higher score indicating better quality of life. Equation: HR_max_ (beats/min) = 220 - age

### Impact of exercise training on plasma BDNF response

**Fig. 3:**
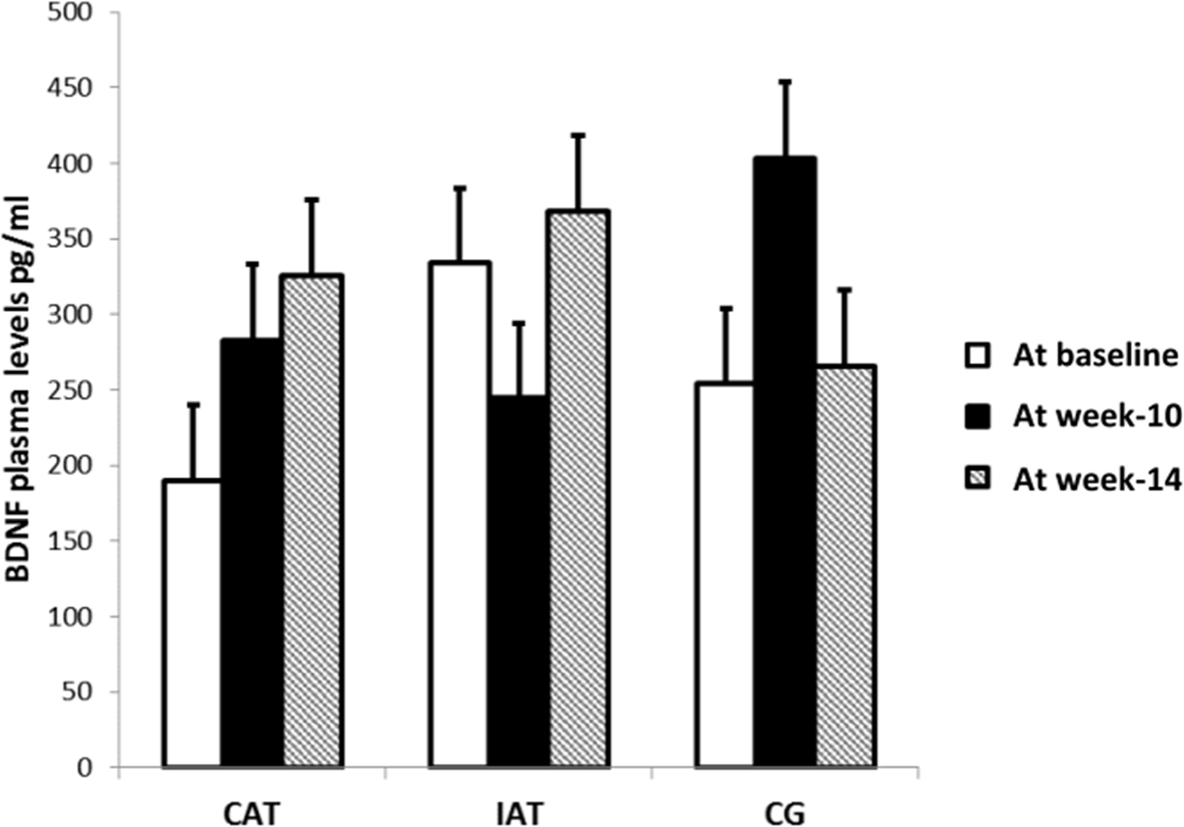
Effect of 9 weeks of aerobic training and 4 weeks of detraining on BDNF plasma level

The effects of exercise modality and detraining period on plasma BDNF levels are shown in Fig. 3. No significant change was observed after 9 weeks of CAT (282.7 pg/ml, 191–467.6) or IAT (244.5 pg/ml, 180.6–380.7) compared to CG (403.7 pg/ml, 205–516.9). There was also no significant difference between the two training groups. At week-14, no significant change was observed in CAT (325.3 pg/ml, 135.6–683.3), IAT (368 pg/ml, 183–631) and controls (265.9 pg/ml, 212.3–481.8) compared to baseline values and week-10.. Similarly, when the same analysis was conducted with outliers, no significant change in plasma BDNF levels was observed after 9 weeks of CAT (305.9 pg/ml, 191–499.6) or IAT (244.5 pg/ml, 180.6–380.7) compared to CG (424.9 pg/ml, 206.6–515.8). At week-14, no significant change was observed in CAT (325.3 pg/ml, 135.6–683.3), IAT (368 pg/ml, 183–631) and controls (287.8 pg/ml, 109–452.7) compared to baseline values and week-10. There was also no significant difference between the three study groups whatever the analysis method. The ANCOVA did not show any significant effect of the considered potential confounders for plasma BDNF level at baseline (i.e.*,* gender, age, MMSE score, aerobic fitness in METs, storage duration of samples, pro-cognitive and antidepressive drugs). No chronic alcohol consumers and smokers were identified among participants. No correlation between plasma BDNF level and MMSE, RAVLT and digit span was found (all *p*. > *0.05*).

### Complementary analyses to identify determinants of plasma BDNF response

In the view of the extreme variability of the plasma BDNF level response within the three study groups and between groups, complementary analyses were computed with the aim to identify explaining determinants of these individual’s response after aerobic-type training. Due to the absence of significant difference between the CAT and IAT after training and detraining, the patients of these two groups were gathered. Thus, among participants, responders were defined as those in which, at least, a 10% increase of plasma BDNF level was measured between week-10 and baseline. This threshold was based on a previous studies that reported a small change of plasma BDNF level after aerobic-type training [53, 54]. Other participants were then considered as non-responders. This was considered in the three study groups because BDNF level is influenced both by physical activity and social interactions [55, 56].

**Fig. 4:**
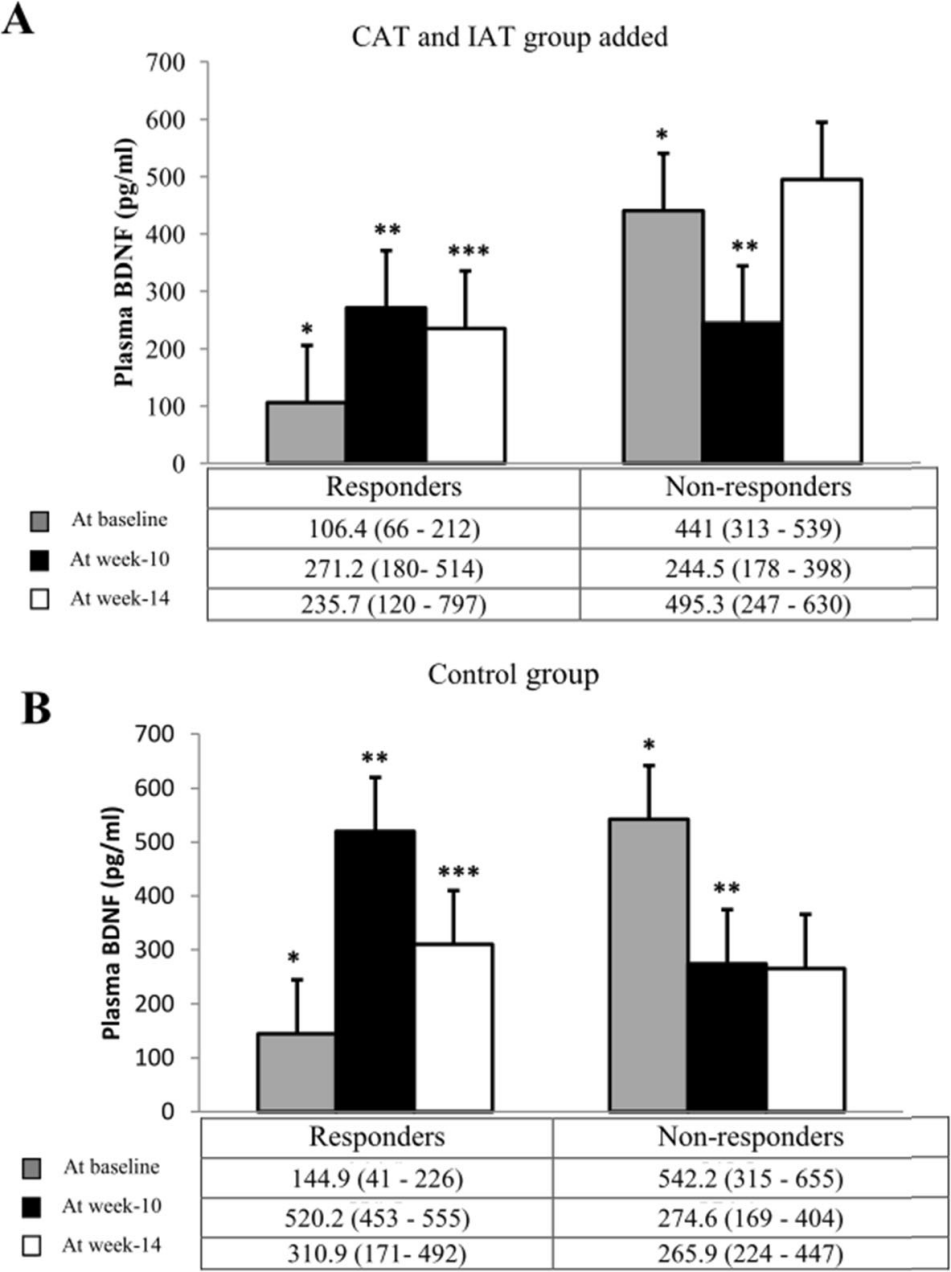
At the bottom, exploratory analysis of plasma BDNF level after 9 weeks of training (week-10) and after a detraining period (week-14) for continuous aerobic (CAT) and interval aerobic training (IAT) group added and control group. Data are presented as median and interquartile range. At the top BDNF plasma level during study *Significant difference between responder and non-responders at baseline (*p* < 0.05); **Significant difference between baseline and after 9 weeks (*p* < 0.05); *** Significant difference between baseline and the detraining (*p* < 0.05)

Among the 31 volunteers enrolled in training groups, 16 were responders (CAT_RESPONDER_ = 9; IAT_RESPONDER_ = 7) and also 10 among the 21 controls. When responders and non-responders’ medical, inflammation profile, cognitive characteristics, and fitness parameters collected at baseline were compared, no significant differences were observed [See Additional file 2]. Figure 4a and b show the evolution of BDNF levels over time in responders compared to non-responders according to the study group. Among responders, it was measured that in those assigned to the two training groups, BDNF response was maintained (*p = 0.9*) between week-10 and week-14 while in controls the plasma BDNF level seems decreased without reach the threshold of significance (*p = 0.07*). In non-responders assigned to the aerobic training sessions, BDNF response also trended upwards between week-10 and week-14 but did not reach the threshold of significance (*p = 0.06*).

At baseline, non-responders had a higher plasma BDNF level compared to responders whatever the group of assignment (*p < 0.01*).

### Aerobic fitness and functional capacity

The impact on aerobic fitness parameters is detailed in Table 2. Compared to control, after 9 weeks of training, the METs was improved by 14.3% in the CAT group (+ 0.6 MET; *p = 0.01*) and 22.7% in the IAT group (+ 1 METs; *p = 0.001*). The MTP was enhanced by 30.8% (*p = 0.009*) and 40% (*p = 0.004*) in CAT and IAT group, respectively. In term of functional capacity, the distance walked during the 6MWT was extended of + 4.7% (+ 28 m; *p = 0.005*) and + 7.2% (+ 36 m; *p = 0.007*) in the CAT and IAT group, respectively. METs, MTP and the distance walked at the 6MWT remained statically unchanged among controls after 9 weeks. No significant difference was measured between IAT and CAT group for METs, MTP, and the distance walked at the 6MWT (all *p > 0.05*).

**Table 2:** Effect of 9 weeks on aerobic fitness and functional capacities in different groups

Characteristic	CAT group, *n* = 14		IAT group, *n* = 17		Control group, *n* = 21	
	Pre - Training	Post - Training	Pre - Training	Post - Training	Pre - Intervention	Post - Intervention
METs	4.2 (3.2–4.9)	4.8 (4.2–5.9)*^a^	4.4 (3.9–4.9)	5.4 (4.3–6.1)*^b^	3.4 (2.9–4.2)	3.9 (2.8–4.5)^a,b^
Maximal tolerated Power (watts)	52 (42–60)	68 (65–79)*^a^	50 (50–66)	70 (60–80)*^b^	40 (35–70)	50 (35–70)^a,b^
Distance 6MWT (meters)	470 (402–494)	492 (465–526)*	430 (370–460)	461 (420–530)*	420 (360–450)	380 (320–475)

Median and rang are provided unless otherwise indicated, *: intra group difference (*p* < 0.05). ^a^: between group difference (*p* < 0.05), ^b^: between group difference (*p* < 0.05). Abbreviations: MET = Metabolic equivalent task; 6MWT = 6 Minutes’ Walk Test

### Cognitive performance

The impacts of the study intervention groups on cognitive performance are presented in Table 3. Globally, there was no significant effect on global cognition and memory tests and sub-tests after the training period. Whilst a significant difference was found in MMSE score between the two training groups (CAT: + 11.1% vs. IAT: − 5.6% – *p = 0.04*), no significant difference was measured for RAVLT, forward digit span and backward digit span from baseline to post-training (all *p > 0.05*). We measured a decrease of attention and calculation in MMSE subtest scores in IAT group (− 50%; *p = 0.03*) and a decrease in MMSE score at the limit to significance threshold in CG (− 9.5%; *p = 0.05*). RAVLT and digit span forward -backward score remained statically unchanged among controls after interactive information sessions. No significant difference was observed between CG and the two-training group.

**Table 3:** Comparison of outcomes measure scores before and after the intervention in different groups

Variables	CAT group, *n* = 14		IAT group, *n* = 17		Control group, *n* = 21	
	Pre - Training	Post - Training	Pre - Training	Post - Training	Pre - Intervention	Post - Intervention
Global cognition						
MMSE, Sub test:						
Orientation	4.5 (4–6)	6 (4.3–7)	5 (4–6)	5 (3–7)	7 (4.8–8)	5 (4–7)
Registration	3 (3–3)	3 (3–3)	3 (3–3)	3 (3–3)	3 (3–3)	3 (3–3)
Attention and calculation	1 (0–4)	1.5 (0–4)	2 (1–3)	1 (0–2)^*^	2.5 (2–4)	2 (1–4)
Free recall	0 (0–1)	0 (0–1)	0 (0–0)	0 (0–0)	0 (0–0)	0 (0–0)
Language	8 (7–8)	8 (7–8)	7.5 (7–8)	7 (7–8)	7.5 (7–8)	7 (7–8)
Visual construction	1 (0.3–1)	1 (0.3–1)	1 (0–1)	0 (0–1)	1 (0–1)	1 (0–1)
MMSE, total score	18 (16–21)	20 (18.3–21)^a^	18 (17–19)	17 (15–21)^a^	21 (17–23)	19 (15–22)
Neurocognitive domain						
Memory:						
RAVLT	22 (13–26)	21.5 (15.5–25)	21 (16–25)	17 (15–24)	19 (18–24)	19 (15–20)
Working memory:						
Forward Digit Span	5 (3–6)	5.5 (4–6)	4 (4–5.3)	5.5 (4–6)	5 (4–7)	5 (4–6)
Backward Digit Span	2 (2–3)	3 (2.3–3)	3 (2–3)	3 (2–3)	3 (2–4)	3 (2–3)

Median and range are provided unless otherwise indicated. *: intra group difference (*p < 0.05*). ^**a**^: between group difference (*p* < 0.05). Abbreviation: MMSE, score: Mini-Mental State Examine, score 0 to 30 with higher score indicating better global cognition; Orientation, score 0 to 10; Registration, score 0 to 3; Attention and calculation, score 0 to 5; Free recall, score 0 to 3; Language, score 0 to 8; Visual construction, score 0 to 1; RAVLT: Rey Auditory Verbal learning Test, score 0 to 75 with higher score indicating better memory; Digit span (Forward and backward), score 0 to 9 with higher score indicating better working memory direct or indirect

### Quality of life

As shown in Table 4, the QoL-AD composite score within the CAT group was improved significantly (+ 5.9%; *p = 0.008*) compared to IAT participants and controls after intervention period. The difference between groups was in favor of CAT group compared to controls (*p = 0.02*). Two domains of the QoL-AD tool were significantly improved. They were humor (*p = +* 50%; *p = 0.02*) and money (+ 50%; *p = 0.01*). No significant difference was observed between the two-training group [See Additional file 3 for QoL-AD total score patient and caregiver].

**Table 4:** Comparison of QoL-AD composite scores, before and after the interventions in different groups

Item QoL-AD	Continuous training group, *n* = 14		Interval training group, *n* = 17		Control group, *n* = 21	
	Pre-training	Post-training	Pre-training	Post-training	Pre-intervention	Post-intervention
(1) Physical health	2 (2–3)	3 (2–3)	2 (2–3)	3 (2–3)	2 (2–3)	3 (2–3)
(2) Energy	3 (2–3)	3 (2–3)	3 (2–3)	3 (3–3)	2 (2–3)	3 (2–3)
(3) Mood	2 (2–3)	3 (2–3)*	3 (2–3)	3 (2–3)	2 (2–3)	2 (2–3)
(4) Living situation	3 (3–3)	3 (3–3)	3 (3–3)	3 (3–3)	3 (2–3)	3 (3–3)
(5) Memory	2 (1–3)	2 (2–2)	2 (2–2)	2 (2–3)	2 (1–2)	2 (2–2)
(6) Family	3 (2–3)	3 (3–3)	3 (3–3)	3 (3–3)	3 (3–3)	3 (2–4)
(7) Marriage	3 (3–4)	3 (3–4)	3 (2–3)	3 (2–3)	3 (2–3)	3 (2–3)
(8) Friends	3 (2–4)	3 (3–3)	3 (3–3)	3 (2–3)	3 (3–3)	3 (2–3)
(9) Self	3 (2–3)	3 (3–3)	3 (2–3)	3 (2–3)	3 (2–3)	3 (2–3)
(10) Ability to do chores	3 (2–3)	3 (2–3)	3 (2–3)	3 (2–3)	2 (2–3)	2 (2–3)
(11) Ability to do things for fun	3 (2–3)	3 (2–3)	3 (2–3)	3 (2–3)	2 (2–2)	2 (2–3)
(12) Money	2 (2–3)	3 (2–3)*	2 (2–3)	3 (2–3)	2 (2–3)	3 (2–3)
(13) Life as a whole	3 (2–3)	3 (3–3)	3 (2–3)	3 (2–3)	3 (2–3)	2 (2–3)
Total score	34 (32–36)	36 (34–37)*^a^	34 (32–35)	34 (32–36)	32 (29–34)	32 (29–36)^a^

Median and range are provided unless otherwise indicated. *: intra group difference (*p < 0.05*). ^**a**^: between group difference (*p < 0.05*) QOL-AD: Quality of Life in Alzheimer’s Disease, total score 13 to 52 with higher score indicating better quality of life

### Feasibility and adverse events

Among the 54 enrolled participants, only 2/54 did not complete the full study program. One because of a flu-like illness episode and the second was injured during a domestic accident.

The 18 sessions composing the endurance training programs were followed by 49/52 participants for global adherence rate of 94.2%. Two individuals did not participate in 1 to 3/18 sessions in the CAT group. One individual did not participate in 2/18 sessions in the IAT group. In CAT, mean training intensity represented 76% of HR_max_ and the workload went from 23 watts during the first training to 48 watts during the last one whereas in IAT, the starting workload was 21 watts (base) and 43 watts (peak), and increased up to 26 watts (base) and 77 watts (peak). The mean exercise intensity was 75% of HR_max_. All patients were able to achieve the desired physical stress. No adverse event induced by the training program including muscle aches was reported.

## Discussion

This randomized study has compared the effect of CAT and IAT in seniors with mild to moderate AD. These two training programs conducted on two sessions per week over 9 weeks did not demonstrate significant changes in plasma BDNF response and were not associated with enhancement in participants’ cognitive performance in this specific population. Compared to controls, CAT and IAT were however effective to improve aerobic fitness parameters and functional capacities with globally no significant difference between them. Better QoL was also reported but only in those engaged in CAT group. This study demonstrated a good adherence rate (94.2%) for both trainings; no serious adverse events and a good tolerance as previously reported by others were measured [57–59].

Previous studies reported a significant increase in peripheral BDNF level after aerobic training in seniors [54]. Albeit globally negative, the present study is, to the best of our knowledge, the first that has investigated a comparative impact of two different types of aerobic-type training (i.e. CAT vs. IAT) on plasma BDNF levels in the specific population of seniors with mild to moderate AD. These findings give however raise to different hypotheses to explain the highly heterogeneous variation in the response to aerobic-type training but also to social and cognitive stimulation as considered in the CG. The workloads we programmed for CAT and IAT were based on a percentage of HR_max_ as reference value and the participant’s physical capacity to perform a maximal effort during IMET. It has been observed that aerobic-type training at a fixed intensity leads to interindividual inhomogeneous metabolic responses [60]. Thus, our method based on global recommendations aiming to improve and maintain health in older adults may not have been sufficiently intense in some participant to induce a significant effect on plasma BDNF production [24, 28, 61]. Several additional factors can however influence the basal synthesis of BDNF at the individual level such as genetic variation, metabolic disorders, and/or inflammatory process and hence the BDNF response to aerobic-type training [62]. For example, some authors consider AD as a metabolic disorder mediated by insulin-resistance at the brain level [63]. The impaired glucose utilization resulted from the accumulation of amyloid plaques combined with, the increased oxidative stress and low grade inflammation, contribute to create a vicious circle that progressively induce and alters cerebral insulin sensitivity and neuronal survival [63, 64]. Acting as a regulatory pathway, plasma BDNF synthesis could be thus upregulated in some people with AD in order to try to counteract and compensate the insulin resistance and neuronal loss, independently of the illness severity and associated treatments [65–68]. The exploratory analysis of the plasma BDNF level conducted in the present study has shown that non-responders had a higher level of plasma BDNF compared to responders at baseline. They also elicited a change towards a decrease in their plasma BDNF levels after training. While it is not possible to clearly attribute the reduction of plasma BDNF level among non-responders to a greater signalization and uptake by the central nervous system after endurance training [15], one possible underlying mechanism would be that BDNF retrogradely crosses the blood–brain barrier to promote the neuronal survival [69–71]. Similar observations and hypotheses have been stated by Dougerthy et al. in pre-clinical stages of AD [72]. This study reported a decrease trend in BDNF levels after 6 months of aerobic exercise that was greater and significant among oldest patients. In the present study, the small sample size and the resulted poor powerful has certainly contributed to limit the ability to detect significant difference in BDNF response.

After aerobic-type training or interactive sessions for CG, no change in MMSE, RAVLT and digit span scores was measured despite variations in plasma BDNF levels (i.e. responder or non-responders). This may suggest that plasma BDNF level is probably not a useful marker for cognitive status, as mentioned by previous studies [73, 74]. It could be also suggested that exists a delayed response between change in BDNF level and improvement in cognitive performance.

Several systematic reviews described aerobic-type training in AD has a light but heterogeneous benefit on patients’ cognition and memory capacities [75–77]. The reasons would be that a minimal duration, frequency and intensity in a short program duration be necessary, to bring about changes in global cognition [76, 78]. For example, Kemoun et al. after 15 weeks of walk (3-weekly, 60-min by session) found an improvement in global cognition while Yu et al. reported not any change in AD patients after 6 months of practice at the rate of 2-weekly 45-min sessions [79, 80]. Another reason would be that multimodal training (i.e., combined aerobic training and strength training) could lead about greater improvement in cognitive function (i.e., memory, executive function, global cognition) and structural brain reserves mediated by more neurological mechanism (e.g., promoting insulin growth factor-1 and vascular endothelial growth factor) than aerobic training alone [76, 77, 81].

Although the present results did not reveal a positive impact on the BDNF response and cognitive performance, the two training protocols provided enough intensity, frequency, and duration to significantly improve volunteers’ aerobic fitness and functional capacities. It is widely agreed that a better aerobic fitness expressed in MET (1 MET = 3.5 ml O_2_/kg^− 1^/min^− 1^) or maximal oxygen uptake (VO_2max_ or VO_2peak_) is associated with lower risk of all-cause mortality, reduced cardiovascular risk factor and useful to maintain functional independence in performing activities of daily life [82–84]. Compared to controls; the increase in METs resulted from CAT (+ 14.3%) and IAT (+ 22.7%) were in the line with the results reported by Sobol et al. [85]. The authors found an increase by + 13% in VO_2peak_ after 16 weeks of moderate to high intensity aerobic exercise (70–80% HR_max_) in mild to moderate AD. The possible reasons explaining the observed improvement would be that an optimal intensity for moderate to maximal aerobic fitness benefits in seniors (i.e., between 70 and 86% of HR_max_), regardless of aerobic training modality, would be needed [27, 86]. It is also known that AD is likely to reduce the functional abilities, with reduced walking speed which may, in turn, lead to progressive loss of functional autonomy and dependency [37, 87]. The lengthening of distance walked in 6MWT measured in participants of the CAT and IAT was of similar extend to which reported by Venturelli et al. and in other studies conducted in patients with AD [88–90]. This also confirms the functional benefits of endurance training in seniors with AD.

Apart from physical and mental health, QoL is also an important component factor to be taken into account when taking care of AD patients [91]. Compared to controls, the beneficial impact on QoL after CAT evokes the key role of aerobic training to favor social interaction and to improve mood and self-esteem [92, 93]. The present findings are consistent with those previously reported [25]. For example, Abd El-Kader et al. showed that 2 months of moderate aerobic exercise improved all SF-36 subscale scores and mood of AD patients [94]. However, the improvement of QoL-AD score was not significant after IAT. QoL-AD composite integrates caregiver score, which can be negatively influenced by caregiver’s burden and patient’s behavioral symptom repercussion [42]. These are factors we did not evaluate. Thus, we cannot conclude that IAT could not have any benefit on patients’ QoL.

Our study has several limitations. Apart from the very small sample size that limit our power to detect significant difference, we cannot formally exclude the positive impact of information sessions acting as social and cognitive stimulation on plasma BDNF synthesis thus masking the possible benefit of aerobic-type training [95]. The intensities programmed in CAT and IAT could have lacked precision to favor homogeneous responses on plasma BDNF production. We cannot also exclude that participants were committed in additional unrecorded physical exercise outside the program which could also be a confounding factor that affected the results. Other limitations include unknown group difference in presence of genetics factors and which could influence the BDNF synthesis such as BDNF gene polymorphism [53, 96].

## Conclusion

This study shows that 9 weeks of CAT and IAT failed to induce significant plasma BDNF response and improve cognitive performance while equally effective to enhance aerobic fitness and functional capacities in senior with mild to moderate AD. CAT had also a beneficial impact on QoL. The highly heterogeneous plasma BDNF response measured among participants suggest the importance of future studies to consider potential confounders and modulator of plasma BDNF production, such as, BDNF polymorphism and other genetic variants. In addition, further researches are also needed to determine whether peripheral plasma BDNF reflect central BDNF in the particular situation of AD.

## Supplementary information

**Additional file 1.** : Detail of analysis of plasma BDNF levels

**Additional file 2.** : Characteristics of “Responders” and “No responders” ‘by group

**Additional file 3.** : QoL-AD scores, patient and caregiver, in different groups, before and after intervention

## Data Availability

The datasets used and/or analyzed during this study are available from the corresponding author on reasonable request.

## Abbreviations

6MWT: 6-min walking test
AD: Alzheimer’s disease
ADL: Activity of Daily Life
Aβ: Amyloid-βeta
BDNF: Brain Derived Neurotrophic Factor
BMI: Body Mass Index
BP: Blood pressure
CAT: Continuous Aerobic Training
CG: Control Group
DBP: Diastolic Blood Pressure
GDS: Geriatric Depressive Symptoms scale
HR: Heart Rate
IADL: Instrumental Activity of Daily Life
IAT: Interval Aerobic Training
IMET: Incremental Maximal Exercise Tests
HR_max_: Maximal Heart Rate theoretical
HR_peak_: Maximal Heart Rate
MET_s_: Maximal Metabolic Equivalent of Task
MMSE: Mini Mental State Examination
MTP: Maximal Tolerated Power
QoL: Quality of Life
QoL-AD: Quality of Life of Alzheimer Disease
RAVLT: Rey Auditory Verbal Learning Test
SBP: Systolic Blood Pressure
