# Supplementary material for: Effect of 9 weeks continuous vs. interval aerobic training on plasma BDNF levels, aerobic fitness, cognitive capacity and quality of life among seniors with mild to moderate Alzheimer’s disease: a randomized controlled trial
Source: Eur Rev Aging Phys Act. 2020 Jan 6;17:2. doi: 10.1186/s11556-019-0234-1 (PMC6945614; doi:10.1186/s11556-019-0234-1)
Supplement: Supplementary file 1 — Additional file 1. : Detail of analysis of plasma BDNF levels [file 11556_2019_234_MOESM1_ESM.doc]

**Additional file 1.** Detail of analysis of plasma BDNF levels.

BDNF levels were measured by Enzyme-Linked Immunosorbent Assay (ELISA) using the BDNF Emax ® ImmunoAssay System (Promega, Madison, WI, USA) according to the manufacturer’s instructions.

A flat-bottom, 96-well, polystyrene microplate (Nunc-Immune™ MaxiSorp™, Cat.# 439454; Thermo scientific Incorporated, Denmark) was covered with 100 µL/well anti-BDNF monoclonal antibody diluted 1:1000 in carbonate buffer (25mM NaHCO3, 25mMNa2CO3, pH 9.7), sealed and incubated overnight at 4◦C. Then, the plate was washed with TBST washing buffer (20Mm Tris-HCl (pH 7.6), 150mM NaCl, 0.05% Tween® 20) and 200µL of 1x Block & Sample buffer were added to each well. The plate was sealed, incubated at room temperature for 1 h, and washed 3 times. Plasma samples (1:2 dilution in 1x Block & Sample) or the BDNF standard curve (1:2 serial dilutions ranging from 500 to 0 pg BDNF/ml) were added to each well (100µL/well), in duplicates. The plate was sealed and incubated for 2 h at room temperature with shaking. After washing the plate 5 times with TBST washing buffer, 100 µL anti-BDNF polyclonal antibodies (diluted 1:500 in 1x Block & Sample buffer) were added to each well, and the plate was sealed and incubated for 2 h at room temperature with shaking. After this step, the plate was washed 5 times, and the anti-IgY horseradish peroxidase conjugate diluted 1:200 in 1x Block & Sample buffer was added (100µL/well). The plate was sealed and incubated for 1 h at room temperature with shaking. The plate was washed for 5 more times and then incubated for 10 min with shaking at room temperature for the color development (100µL of TMB One solution per well). The reaction was interrupted by adding 100µL/well 1N HCl and absorbance was measured at 450 nm on the Biorad instrument EVOLIS®. All samples and standards were measured in duplicate. When the BDNF value was out of the standard curve value, we performed a new evaluation with a ¼ dilution, in duplicate. The means of the duplicates was used for statistical analyses.

A standard curve has been done for every plate. Each standard curve has a coefficient of determination about r^2^=0.999.
