# Supplementary material for: Effect of 9 weeks continuous vs. interval aerobic training on plasma BDNF levels, aerobic fitness, cognitive capacity and quality of life among seniors with mild to moderate Alzheimer’s disease: a randomized controlled trial
Source: Eur Rev Aging Phys Act. 2020 Jan 6;17:2. doi: 10.1186/s11556-019-0234-1 (PMC6945614; doi:10.1186/s11556-019-0234-1)
Supplement: Supplementary file 2 — Additional file 2. : Characteristics of “Responders” and “No responders” ‘by group [file 11556_2019_234_MOESM2_ESM.docx]

| **Additional file 2.** Characteristics of “Responders” and “No responders” ‘by group. | | | | |
| --- | --- | --- | --- | --- |
| **Characteristic** | **(CAT + IAT), n=31** | | **CG, n=21** | |
|  | **Responders’ n=16** | **No responders’ n=15** | **Responders’**  **n=10** | **No responders’ n=11** |
| Age (years) | 79 (72-84) | 76 (70-81) | 79.5 (75-83) | 9 (74-85) |
| Female, *n* | 13 | 10 | 5 | 6 |
| Male, *n* | 3 | 5 | 5 | 5 |
| Weight(kg) | 63 (54-69) | 61 (51-72) | 72 (62-74) | 60 (58-80) |
| Height (cm) | 166 (162-170) | 164 (163-169) | 172 (163-176) | 69 (164-172) |
| Body mass index (kg/m^2^) | 21.6 (21-25) | 22.2 (21-24) | 23 (22-25) | 23.9 (21-26) |
| Education level (years) | 7 (7-10) | 7 (7-10) | 7 (7-7) | 7 (7-9) |
| Alzheimer duration (Years) | 2.5 (2-3) | 2 (1-4) | 2.5 (1.2-3) | 3 (1.8-8.3) |
| Nursing home residing, *n* | 2 | 1 | 2 | 4 |
| Home-living population, *n* | 14 | 14 | 8 | 6 |
| **Medicine** |  |  |  |  |
| Antihypertensive treatment, *n* | 10 | 9 | 7 | 6 |
| Anti – Alzheimer’s treatment, *n* | 9 | 10 | 6 | 3 |
| Antidiabetic, *n* | 4 | 2 | 2 | 2 |
| Depression treatment, *n* | 1 | 2 | 3 | 2 |
| Statins, *n* | 2 | 4 | 0 | 2 |
| **Inflammatory marker** |  |  |  |  |
| C-reactive protein | 0.6 (0.6-0.9) | 0.6 (0.6-1.7) | 0.9 (0.6-2.3) | 1 (0.8-1.8) |
| **Cardiorespiratory parameters** |  |  |  |  |
| MTP | 50 (50-62) | 56 (43-65) | 55 (40-69) | 35 (27.5-55) |
| METs | 4.2 (6.6-4.7) | 4.4 (3.6-4.9) | 3.8 (3.2-4) | 3.1 (2.7-3.7) |
| 6MWT | 447 (404-465) | 446 (360-533) | 433 (419-450) | 390 (330-436) |
| **Cognitive performance** |  |  |  |  |
| MMSE | 18.5 (16-19) | 18 (17-20) | 19.5 (17-21) | 21 (18-23) |
| RAVLT | 23 (14.8-26) | 18 (15-23.5) | 20.5 (18-23.8) | 19 (16-23.5) |
| Forward Digit Span | 5 (3.5-6) | 4 (4-6) | 5.5 (4.3-7) | 5 (4.5-6.5) |
| Backward Digit Span | 2 (2-3) | 3 (2-3) | 3.5 (2-4) | 3 (2-3.5) |
| Median and rang are provided unless otherwise indicated. Abbreviations: MTP: Maximal Tolerated Power; METs; Equivalent Metabolic Task ; MMSE: Mini Mental State Examination; RAVLT : Rey Auditory Verbal Learning Test; 6MWT : 6 Minutes’ Walk Test. | | | | |
