# Supplementary material for: Effect of 9 weeks continuous vs. interval aerobic training on plasma BDNF levels, aerobic fitness, cognitive capacity and quality of life among seniors with mild to moderate Alzheimer’s disease: a randomized controlled trial
Source: Eur Rev Aging Phys Act. 2020 Jan 6;17:2. doi: 10.1186/s11556-019-0234-1 (PMC6945614; doi:10.1186/s11556-019-0234-1)
Supplement: Supplementary file 3 — Additional file 3. : QoL-AD scores, patient and caregiver, in different groups, before and after intervention [file 11556_2019_234_MOESM3_ESM.docx]

| **Additional file 2.**  QoL-AD scores, patient and caregiver, in different groups, before and after intervention. | | | | | | | |
| --- | --- | --- | --- | --- | --- | --- | --- |
| **Total score** | **Continuous training group , *n*=14** | | **Interval training group, *n*=17** | | **Control group, *n*=21** | | |
|  | **Pre-training** | **Post-training** | **Pre-training** | **Post-training** | **Pre-intervention** | | **Post-intervention** |
| QoL-AD patient | 36 (31-38) | 37 (4-39) | 34 (32-37) | 35 (34-38) | 33 (31-35) | 35 (30-37) | |
| QoL-AD caregiver | 31 (29-35) | 33 (31-40)* | 31 (28-35) | 32 (26-36) | 29 (26-322) | 31 (28-33) | |
| Median and range are provided unless otherwise indicated, *: intra group difference (p <0.05). QOL-AD: Quality of Life in Alzheimer's Disease, total score 13 to 52 with higher score indicating better quality of life. | | | | | | | |
